# Alcohol and Violence in the Emergency Room: A Review and Perspectives from Psychological and Social Sciences

**DOI:** 10.3390/ijerph10104584

**Published:** 2013-09-27

**Authors:** Oulmann Zerhouni, Laurent Bègue, Georges Brousse, Françoise Carpentier, Maurice Dematteis, Lucie Pennel, Joel Swendsen, Cheryl Cherpitel

**Affiliations:** 1 Laboratoire Interuniversitaire de Psychologie, Personnalité, Cognition, Changement Social, UFR SHS, 1251 avenue Centrale, BP 47, 38040 Grenoble Cedex 9, France; E-Mail: laurent.begue@upmf-grenoble.fr; 2 CHU Clermont Ferrand, Urgences Adultes, 28 Place Henri Dunant BP 69, 63003 Clermont-Ferrand Cedex 01, France; E-Mail: gbrousse@chu-clermontferrand.fr; 3 UFR Médecine, Université Clermont 1, Place Henri Dunant, Clermont-Ferrand F-63001, France; 4 UFR Médecine, Université Clermont 1, EA 7280, Clermont-Ferrand F63001, France; 5 Centre hospitalier universitaire de Grenoble, CHU de Grenoble BP 217 38043 Grenoble cedex 09, France; E-Mail: fcarpentier@chu-grenoble.fr; 6 INSERM U1042, Grenoble F-38042, France; E-Mail: mdematteis@chu-grenoble.fr; 7 Faculté de Médecine, Université Joseph Fourier, Grenoble F-38042, France; E-Mail: lpennel@chu-grenoble.fr; 8 CHU, Hôpital Michallon, Addictologie, Grenoble F-38043, France; 9 INSERM U836, Equipe 10, Grenoble F-38042, France; 10 CNRS UMR 5287, INCIA, Institut de Neurosciences cognitives et intégratives d’Aquitaine, Université Victor Segalen, Bordeaux 2, 146 rue Léo Saignat, 33076 Bordeaux cedex, France; E-Mail: joel.swendsen@psycho.u-bordeaux2.fr; 11 Alcohol Research Group, Public Health Institute, 6475 Christie Avenue, Suite 400, Emeryville, CA 94608, USA; E-Mail: ccherpitel@arg.org

**Keywords:** alcohol, violence, injury, emergency room, aggression, social cognition

## Abstract

Our objective is to present a focused review of the scientific literature on the effect of alcohol consumption on violence related-injuries assessed in the emergency room (ER) and to show how psychological and behavioral sciences could lead to a better understanding of the factors contributing to alcohol-related injuries in the ER. We retrieved published literature through a detailed search in Academic Search Premier, MEDLINE with Full Text PsycARTICLES, Psychology and Behavioral Sciences Collection, PsycINFO, PUBMed and SocINDEX with Full Text for articles related to emergency rooms, medical problems and sociocognitive models addressing alcohol intoxication articles. The first search was conducted in June 2011 and updated until August 2013. Literature shows that compared to uninjured patients; injured ones have a higher probability of: (i) having an elevated blood-alcohol concentration upon arrival at the ER; (ii) reporting having drunk alcohol during the six hours preceding the event; and (iii) suffering from drinking-related consequences that adversely affect their social life. The main neurocognitive and sociocognitive models on alcohol and aggression are also discussed in order to understand the aetiology of violence-related injuries in emergency rooms. Suggestions are made for future research and prevention.

## 1. Introduction

### 1.1. Need for New Perspectives in ER Research

Alcohol misuse is associated with approximately 60 distinct forms of disease and injury [1] and it is one of the leading causes of death among individual between 12 and 20 years, mainly due to unintentional injuries, homicide, and suicide [2,3]. Alcohol has been shown to play a causal role in aggressive behavior in animals and humans [4,5]. Worldwide estimations show that among all work disabilities due to drinking, 12% can be ascribed to intentional injuries [6]. The main symptoms of alcoholic intoxication are an impaired sense of balance and coordination, a drop in attention, and slow reaction times. These factors increase the risk of injury, as in road accidents (in the United States, blood alcohol concentrations (BACs) of 0.8%, 1.0%, 1.5%, and 2.0% have been found to be respectively associated with a 2-, 7-, 10-, and 20-fold increase [7]. But the role of drinking in triggering violent behaviors is difficult to assess because these behaviors occur at times when it is hard to measure them. Despite this measurement difficulty, data on the impact of alcohol on injury is now widely available. According to a report published by the WHO, 3.2% of the World’s deaths every year can be ascribed to alcohol (*i.e.*, 1.8 million deaths), 13.7% of which are due to deliberately inflicted injuries. 

The emergency room (ER) is a unique place for studying the relationship between drinking and physical injury, because this is where injured people often first come in contact with the treatment system. In the United States, 17% of the yearly ER visits are related to an injury following a violent act [8]. In France, 16.5% to 37.5% of all injuries seen by emergency-room staff are linked to drinking [9,10]. Most studies on injuries due to violence have been conducted in hospital ERs, since this is usually the place where first treatment is given [11]. In the case of death, blood-alcohol content is systematically measured by a forensic physician, although no systematic screening for alcohol use has been set up in ER procedures. The first study aimed at linking drinking to injury dates back to 1969 [12]. Since then, a large body of research has been conducted on this issue, and extensive reviews have been published. Among the many relations uncovered, the two most robust are the link between drinking and accidents involving an injury [11], and the link between drinking and injury resulting from violence [13]. However, prevention outcomes resulting from ER hospitalization are scarce in the literature on emergency medicine. Nonetheless, we think that focusing on prevention and intervention on patients hospitalized in ER for violence and alcohol-related injuries would have substantial health improvements by eventually reducing the proportion of patients who are hospitalized in ER for alcohol-related injuries under alcohol intoxication. 

Since Weschler’s [12] study, very little has changed in terms of prevalence of violence-related injuries while intoxicated in ER. As Table 1 shows, percentages of violence-related injuries are quite similar across time, with mean positive BAC for patient with violence-related injuries being as high as 48.3% in 2007 [14]. In 2012, alcohol was involved in 62.9% of violence-related injuries [15]. Interventions aimed at reducing alcohol consumption among patients in ER have been existing for more than 20 years [16] and some have demonstrated their efficiency, such as Motivational Interviewing [17,18] while other types of intervention seem to have little to no effect at all on reducing alcohol consumption [19]. To our knowledge, no intervention has been especially targeted at intoxicated patients with violence-related injuries. Since alcohol drinking is one of the main factors of violence-related injuries and that the emergency department is the first place where people come to treat their injuries [20], the aim of this review was to propose suggestions for future research on violence-related interventions in the emergency department. 

Research on violence-related injuries in ER is concerned with methodological and theoretical issues, including how contextual behavioral variables can moderate the link between alcohol consumption and violence-related injuries. To our knowledge, little has been done to address these methodological issues. For example, the samples found in an emergency room might not be representative of the general population. Studies have shown that patients in ER had often been questioned more than once (3.6%), which means that certain individuals were over-represented [21]. This problem is seldom taken into account, even though it seems to be present in most studies on the alcohol-injury link. Accordingly, in a study on 988 patients 91 of the patients had already been to the emergency room for a problem related directly or indirectly to alcohol. Among these 91, six had sought help for an alcohol-related problem during the 12 months prior to ER admission, and 10 went back a second time within six months after the first visit [22]. The fact that a substantial proportion of patients went back a second time in the ER leads us to think that most of the admission in the ER that are related to injuries caused by alcohol consumption could be in fact more due to behavioral causes than purely pharmacological causes. Potential psychological profiles may then promote the future occurrence of alcohol-related problems, thereby increasing the risk of another hospitalization. ER studies thus have the limitation of examining different types of populations while considering it as a single one (*i.e.*, individuals suffering from chronic or alcohol problems and with various psychological and sociodemographic profiles). ER research would benefit from experimental research on alcohol and aggression in which these aspects may be controlled in a laboratory setting. Recommendations to take into account behavioral variables has already been done in other papers on violence-related injuries in ER [11,13,14], but we haven’t been able to find a single study that takes these aspects into consideration. In fact, the effects of alcohol on human cognitive and behavioral processes are widely documented and could be used to build prevention programs for patients admitted for violence-related injuries in the ER. Generally, research on behavioral intentions has shown that behavior is more likely to be changed when there has been an intervention because they have an impact on motivational change which has an impact on behavioral change [23] and motivation to change has been shown to be related to behavioral change [24].

Therefore, we think that research in alcohol cognition and social sciences could provide valuable insights and perspectives to develop new methods or intervention dedicated to violence-related injuries and improve the existing interventions. Our review will address this issues by providing perspectives for intervention and prevention from psychological and social science research on intoxicated aggression.

### 1.2. Research Methodology

#### 1.2.1. Selection of Studies

We first conducted a bibliographical research of quantitative studies, narrative reviews and meta-analyses that examined and reported the association between violence-related injuries and alcohol consumption in emergency services. Second, we considered only quantitative studies and reviews on behavioral and social psychological models of alcohol cognition. In this paper, violence-related injuries refer to injuries resulting from intentional causes, compared to injuries resulting from unintentional or accidental causes. Articles focusing on injuries other than violence-related injuries were excluded. Thus, in the ER literature, we included studies in which patients’ alcohol consumption, drinking behavior and intentional injuries were recorded. Only studies with probability sampling (*i.e.*, in which all times and days of the week are equally represented) were included in this review.

Since no longitudinal studies including these three aspects (ER, alcohol and violence) were found, only cross-sectional studies were included. In the behavioral and the social psychological literature, we included experimental studies and reviews examining the role of implicit and explicit cognition, expectancies toward alcohol, personality and attributional processes in aggressive behavior and alcohol consumption. Observational and qualitative studies were not included.

#### 1.2.2. Participants and Patients

All participants in the psychological literature and patients in the ER literature were 18 years-old or older, except in one study in which they were 15 and older [25]. Participants in the Social Psychological Literature were all over 18 years old.

#### 1.2.3. Outcome Measurements

In the ER studies, alcohol intoxication was measured both by self-report and/or by Blood Alcohol Concentration (BAC). BAC was measured directly by blood analysis, or estimated indirectly by a breath analysis, saliva or urine analysis. Self-report of alcohol consumption consists in the patient reporting alcohol consumption 6 h before the injury. In the social psychological literature, research strategy included behavioral, self-report and implicit measures.

#### 1.2.4. Electronic Search Strategy

Studies included in this review were drawn from the English-language scientific literature from 1960 to August 2013, except for one article [9], which was in French. We limited our search to peer-reviewed journals only. We searched Academic Search Premier, MEDLINE with Full Text PsycARTICLES, Psychology and Behavioral Sciences Collection, PsycINFO, PUBMed and SocINDEX with Full Text, supplemented with hand searches of key journals and reference lists of identified papers and key publications for more recent publications. The search strategy used the following two sets of terms: 

Emergency Services Literature Strategy: (Alcohol OR Alcohol Consumption OR Alcohol Drinking) AND (Emergency Room OR ER OR Emergency Services OR ES OR Emergency Department OR ED) AND (Violence OR Violence-related Injuries).

Social Psychological Literature Strategy: (Alcohol OR Alcohol Consumption OR Alcohol Drinking) AND (Aggressive Behavior OR Aggression) AND (Cogn*OR Psych*OR Neuro*) AND (Personality OR Automaticity).

The first author led the electronic search and retrieved all studies corresponding to the mentioned keywords and prescreened the more relevant ones in both fields. A first screening allowed us to exclude studies for which the title and/or the abstract was not relevant to the fields of alcohol and violence in emergency room and/or sociocognitive models of alcohol and aggression. If the article could not be excluded on the basis of the title/abstract, the full paper was sent to the other authors for additional screening and review, according to their field of expertise. A study was included when all authors reached consensus on its relevance in his/her field.

#### 1.2.5. Results of the Electronic Search

The search strategy resulted in 354 results for the ER literature and 544 results for the Social Psychological Literature. The number of articles was reduced to 90 in ER literature and to 131 in the Social Psychological Literature after first screening and suppression of duplicates. 

After consensus was reached, the number of articles was reduced to 52 for the ER literature and 74 for the Social psychological Literature. All papers in this review were included following the electronic search, except for reference [26] which has been spontaneously suggested by one of the author.

## 2. Effects of Alcohol on Violence-Related Injuries in Emergency Rooms

### 2.1. General Statistics

Alcohol has an overall impact on the probability of being injured. Alcohol at the time of ER arrival is measured in terms of blood-alcohol content, often using a breathalyzer or by taking a blood or urine sample. Drinking before arrival is measured via self-reports covering the six hours before arrival [11,14]. On average, patients admitted to the ER are more likely to report social consequences of alcohol drinking, score higher on self-report measures of alcohol dependence [27] and report more frequent episodes of heavy drinking than the general population [28]. A positive link exists between the occurrence of an injury and: (i) the probability of having a positive alcohol-test result, (ii) self-reported drinking within the past six hours, (iii) suffering from alcohol-related disorders and (iv) having been in a fight before arriving at the ER. These relationships have been found is numerous studies [11,13,29]. Controlling for age, gender, and drinking habits, Cherpitel and colleagues [30] noted that alcohol drinkers had a significantly higher risk of being admitted to the ER for an injury than for any other medical or surgical condition. A higher prevalence of alcohol consumption prior to the event has been found for injuries resulting from violence (those arising from intentional causes) than for injuries resulting from other (unintentional) causes. Table 1 shows the studies reporting the prevalence of positive BAC and/or self-reported consumption within 6 h prior to the event, which used representative samples of patients. In all studies prevalence estimates were compared between violence-related injuries and non-violence related injuries. Data in this table do not distinguish between injuries sustained by perpetrators and injuries sustained by victims. For violence-related injuries, large proportions on both measures are found, ranging from 17% in the United States to 70% in South Africa and Scotland for positive BAC, and from 36% in Brasil to 87% in Canada for self-report. In all studies those with violence-related injuries are significantly more likely to be BAC positive and to report drinking prior to the event than those sustaining injuries from other causes. 

Patients with violence-related injuries are significantly more likely to be positive for BAC and self-reported drinking prior to the event than those sustaining injuries from other causes. The magnitude of these differences is substantially greater than differences found between injured and non-injured patients for BAC and self-report (Table 1). 

BAC and self-reported drinking are both moderately associated with ER admission for an injury, although contextual variables play a modulating role here. The alcohol/violence relationship has been observed in several countries and adjusting for various socio-economic variables (Table 1). This leads us to think that the impact of alcohol on ER hospitalization go beyond its pharmacological effects. 

Moreover, injuries linked to violence and injuries caused by accidents occurring under the influence of alcohol are clearly separate phenomena. Accordingly, Borges, Cherpitel, and Mittleman’s [31] study conducted on 961 patients in the United States and Mexico showed that the estimated risk of having to go the ER for an injury after consuming alcohol was greater for a violence-linked injury than for an accidental one. These results are corroborated by former studies [32]. Patients with violence-related injuries are more likely to be regular drinkers than those with non-violent injuries [7,33]. In a 1994 review of 11 studies on violence-related injuries handled in the ER, Cherpitel [13] showed that 55% of the patients reported having consumed alcohol in the six hours before the violence-linked injury, versus only 27% for injuries due to other causes. Violent behavior leading to physical injury is usually strongly linked to drinking. In a study, for example, 12% of all alcohol-related health problems were due to deliberately inflicted physical injuries, and alcohol addiction was often found among individuals admitted to the ER for a violence-related injury. For both injured and non-injured patients, more frequent episodes of drunkenness as well as more harmful consequences of alcohol on social life were reported [34]. Generally speaking, patients admitted to a hospital ER who have both a positive BAC and reported having drunk alcohol during the six hours preceding the event are more likely to have a violence-related injury [7,12,35,36]. Despite the association between alcohol consumption and violence, most drinking occasions do not produce aggressive behavior. For example, according to teenager self-reports, there are between 2 and 10 fights per 1,000 drinking occasions [37]. The relationship between alcohol consumption and aggression is conditioned by other variables, among which cultural factors are important. 

### 2.2. International and Cultural Comparisons

The alcohol-violence link varies significantly across cultures, and is stronger in some countries (e.g., South Africa) than in others (e.g., Canada) [38]. These variations are related to differences in drinking patterns, norms, beliefs and expectancies about intoxicated behavior [39]. A study in 13 European countries showed that alcohol-related violence was highest in places were drinking often leads to intoxication [37,40]. The strongest association was observed among Nordic countries, and the weakest relationship was observed in Southern European countries where intoxication is less prevalent. Regarding homicide, according to estimations based on data recorded between 1950 and 1995, a liter increase in alcohol consumption *per capita* leads to an increase in rates of violence of 5.5% in Southern Europe, 8.5% in Central Europe, and 12.4% in Northern Europe [41]. 

Drinking modes vary across countries. As a general rule, patients admitted in emergency rooms have the highest intoxication rate (positive blood-alcohol-test results) on weekends, especially at night. However, this difference has only been found in so-called “dry” countries (*i.e.*, countries where everyday drinking is uncommon and people become intoxicated more often or more quickly when alcohol is consumed). In “wet” countries, on the other hand, where alcohol drinking is part of people’s daily habits (for example, wine is consumed at mealtime), the blood-alcohol content does not differ according to the day the patient arrives at the emergency room [42].

## 3. Is Alcohol a Cause or a Consequence of Violence-Related Injuries?

It appears quite clearly that injuries leading to hospitalization are often linked to drinking. Although drinking at a particular time is the principal factor in the occurrence of an injury, other alcohol-related factors are also reliable predictors of violence-linked injury. Throughout this section, we will discuss various aspects of alcohol-related cognitions and personality variables that could account for the differences in the levels of intoxications between violence-related injuries and other injuries in the ER (Table 1). 

Currently, the links between alcohol and violence are mainly assessed by epidemiological studies. ER studies have found substantial proportions of those who report drinking prior to injury to also report a causal association of their drinking with injury [7], and this and other contextual data on drinking during the injury event would inform tailored interventions for patients. Also, results in Table 1 suggest that violence is systematically associated with greater levels of alcohol intoxication, thus pointing to a strong relationship between alcohol and aggressive behavior. We will discuss whether one factor is causing the other, however we should note that in both cases (*i.e.*, alcohol causing violence or individuals showing personality traits related to violence being more prone to consume alcohol) this could lead to more patient-specific and adapted interventions and eventually to lower alcohol-related injuries in the ER. 

Alcohol is usually considered as the cause for violent behavior and violence-related injuries. For example, in a 2005 meta-analysis on a sample of 17,708 patients from hospitals in seven countries, Cherpitel, and colleagues [7] showed that the fact of having consumed alcohol had a major impact on the occurrence of an injury. The amount of violence-related injury that could be avoided if alcohol had not been consumed was 43%. For example, Vinson and collaborators [43] showed, in a sample of American patients, that persons who had drunk alcohol were ten times more likely to have a violence-related injury than those who had not. Borges and colleagues [44] also showed that the best predictor of injury caused by violent acts is drinking alcohol in the hours prior to arrival at the ER. Epidemiological studies thus seem to show mixed results concerning the magnitude of the link between alcohol and aggression and only provide correlational data.

Experimental psychology has made a unique contribution to the issue of the alcohol-violence link by conducting laboratory studies on how doses of alcohol ingested by human volunteers affect their aggressive reactions [45].

### 3.1. How Neurobiological Effects of Drinking Influence Aggression?

It has been shown that in order to explain the effects of chronic alcoholism on aggression, important factors such as nutritional deficit induced by excessive alcohol consumption, sleep deprivation, impairment of neuropsychological functioning, or enhancement of psychopathological disorders could be considered as relevant variables. These aspects have important consequences on how one processes situational cues and engages in social interactions. In fact, all models of the alcohol-aggression link imply that it is (1) mediated by cognitive and emotional states and (2) may be modulated by chronic knowledge structures [46].

According to Steele and Josephs, alcohol causes excessive social behaviors indirectly by restricting cognitive capacity and leading to a psychological myopic state. Alcohol myopia theory (AMT) is defined as a ‘state of shortsightedness in which superficially understood, immediate aspects of experience have a disproportionate influence on behaviors and emotions [47]. Various studies show that intoxicated people no longer have the prerequisite processing skills to attend to all of the multiple cues involved in social behavior [48]. Thus, according to AMT, aggression is not necessarily the outcome of alcohol consumption if nonaggressive reactions are salient after a provocation. 

Steele and Josephs [47] posit that individuals’ social behaviors are affected by two kinds of cues: those that instigate a behavior (provoking cues) and those that constrain a behavior (inhibitory cues). Situations in which both provoking (e.g., provocation) and inhibitory cues (e.g., fear of the consequences of a fight) are present are referred to as inhibition response conflict, because the inhibitory cue to suppress action operates in opposition to the provoking cue to act. According to AMT, alcohol consumption suppresses inhibitory cues; thus, intoxicated individuals are more likely to act on their provoking cues than are sober individuals. An intoxicated person may therefore not correctly perceive the reasons for other people’s behavior, making the actions of others appear more provocative than they would to a sober perceiver. 

Recent neuropsychological studies on the mediator and moderator status of executive cognitive functioning (ECF) are compatible with Steele and Joseph’s hypothesis on alcohol myopia and Hull’s [49] self-awareness model. ECF is a subset of cognitive capacities associated with prefrontal cortex encompassing a variety of higher order cognitive abilities such as attention, abstract reasoning, organization, mental flexibility, planning, self-monitoring, and the ability to use external feedback to moderate personal behavior [50]. Many studies indicate that ECF is deficient among perpetrators of aggression [51,52,53,54] and is disrupted by alcohol consumption [55,56,57]. Direct empirical tests of a mediational model shows that alcohol’s pharmacological properties facilitate aggression by altering ECF [58,59,60]. Moreover, ECF also moderates the alcohol-aggression link: individuals who have a low level of ECF in a sober state react more aggressively when they drink alcohol [57,58,59,60,61].

The Alcohol Myopia Theory is useful for decreasing intoxicated violence. If an intoxicated person’s attention is distracted away from a provocative stimulus, a reduction of aggression is observed. According to Giancola [62], there are various mechanisms by which distraction might decrease violence-related injuries: the reduction of negative affect and anger, the reduction of cognitive rumination, an increase of self-awareness, and a possible increase of empathy by distracting the individual from provocation. For example, Heppner *et al*. [63] employed an effective technique of aggression reduction by distracting individuals from provocation by encouraging them to focus on the very simple details of eating a grape! Another technique that may be used to limit intoxicated aggression may rely on the inhibitive consequences of self-awareness: the mere increase of self-awareness by putting a mirror in a room is enough to suppress aggression toward others [64]. 

The AMT and the related self-awareness perspective are cognitive models explaining how pharmacological properties of alcohol influence information processing. However, in some cases, alcohol may influence cognitive processes and behavior for reasons that have nothing to do with the psychopharmacological effect of ethanol on the brain. For example, people drove more recklessly in a driving simulator when led to believe that they had just consumed alcohol [65]. These social-cognitive models of alcohol-related aggression are developed in the following sections.

### 3.2. Social-Cognitive Explanations of Alcohol-Related Aggression: Do Beliefs about Alcohol Influence Aggression?

However, the main current social cognitive explanations of the effect of acute alcohol consumption on aggressive behavior are especially relevant to explain acute effects of alcohol, rather than its chronic effects. It has yet been shown that it is the acute effects of alcohol that have the largest impact on aggressive behavior [66,67,68,69]. 

However, many other studies suggest that alcohol may provide an attributional excuse to engage in socially unconventional or prohibited acts. According to the attributional perspective, expectancies operate as an explicit belief that influence the decision-making process. Drinkers simply expect other people to tolerate their antinormative behavior (*i.e.*, behavior that goes against shared social norms) if it can be attributed to alcohol or that alcohol will help them in performing aggressive acts. A premise of the attributional perspective is that one expects alcohol to have specific consequences on oneself and others [70]. The central construct of expectation reflects the representation in memory of an individual’s acquired knowledge (information, encoding, schema, script) regarding the consequences of definite behavior in different contexts by direct experience or vicariously [71]. In alcoholism, expectancies are viewed as major proximal determinants of drinking behavior and as a mediator of many of the other psychological and pharmacological influences [72].

Indeed, beliefs about the effects of alcohol also play a role in the occurrence of violence-linked injuries and could explain the differences in behaviors following alcohol consumptions. Gogineni and colleagues [73] showed with a sample of American ER patients that individuals with a high blood-alcohol level who believed that alcohol makes one less cautious and more careless, had a stronger tendency to be victims of aggression. In contrast, those who thought that alcohol made them more aggressive had a lesser chance of being assaulted. According to sociocognitive theories, beliefs about the effects of drinking are a critical factor in determining the behavior exhibited after drinking. Once under the influence of alcohol, individuals act in accordance with their expectations [61]. Moreover, using experimental methods, Levinson and colleagues showed that alcohol increased aggressive behavior (measured by the frequency and duration of electric shock during a competitive task) in people holding beliefs that alcohol increases aggressive behavior [74].

In another area, theories of embodied cognition showed that cognitive processes are shaped by the aspects and functions of the human body [75] due to links between semantic memory and bodily experience and social interaction [76]. For example, experimental research showed that weight was positively linked with aggression while being intoxicated because greater weight is linked to feelings of importance in semantic memory and that it would provide an advantage if one’s has to engage in physical conflict [77]. Moreover, the influence of weight on alcohol-related aggression was only observed in male participants. 

### 3.3. Contributions from Implicit Social Cognition: The Model of Automaticity

Automaticity refers to a process that is unintentional (the individual does not start the process by an act of will), uncontrollable (the process cannot be stopped), efficient (consume a minimal attentional resource), and occurs outside awareness [78]. For instance, social knowledge is automatically (implicitly) activated in memory during the natural course of perception, and this without people’s awareness or intention. In the implicit social cognition perspective, studies showed that drinking alcohol in not even necessary to trigger aggressive behavior. Mere thoughts about alcohol can activate aggression-related concepts and consequently facilitate aggressive behaviors. Results from experimental social psychology show that mere exposure to alcohol-related cues can increase aggressive thoughts and behaviors: in two studies, Subra and colleagues [79] showed that participants exposed to alcohol cues and weapon cues automatically increased aggressive thoughts (measured with reaction time when asked to categorize aggression-related words) and aggressive behavior toward the experimenter compared with participants exposed to neutral cues. The effect of alcohol cues on aggressive thoughts was just as strong as the effect of weapon cues on aggressive thoughts.

From these results, it seems that a causal link exists which implies that drinking alcohol, in conjunction with other factors (*i.e.*, cognitive variables) could generate aggressive behavior. However, that does not disprove alternatives explanation in which alcohol is used as a facilitator for committing violent acts or as an a posteriori excuse for aggressive and delinquent behavior. Nevertheless, evidence for this aspect is scarce and usually weak [80]. 

Eventually, it appears that alcohol alone is not enough to trigger aggressive behavior as in the experimental studies conducted so far, subjects led to drink alcohol only rarely committed acts of aggression, and only did so if they were first provoked by the confederate (*i.e.*, an actor working with the experimenter but pretending to be a participant) [81,82]. Generally speaking, meta-analyses on these studies have concluded that there is a linear causal effect of alcohol on aggressive conduct in both men and women [83]. 

Theories related to implicit social cognition of alcohol could be useful in assessing attitudes toward alcohol in patients who had been admitted for alcohol-related injuries due to violence. For example, implicit social cognition measures have shown good predictive power for affective association with alcohol use and misuse in alcohol-dependent patients. More interesting, implicit measures explained a significant portion of variance and showed better predictive value compared to self-reported measures [84]. In a sample of patients with alcohol problems, De Houwer and colleagues showed that heavier drinkers had more arousal-related associations with alcohol (*i.e.*, they associate alcohol with the sensation of “wanting”) [85]. In another study, implicit associations in memory uniquely predicted intention concerning 30 day alcohol consumption [86]. For example, implicit social cognition tools could be used to assess implicit attitudes towards alcohol and violence in ER patients to predict their odds of demonstrating aggressive behavior when intoxicated with alcohol.

### 3.4. Interaction with Personality Variables: Does Personality Explains Alcohol-Related Aggression?

Despite the acknowledged relationship between alcohol consumption and aggression, alcohol is neither a necessary nor a sufficient cause of aggression. For example, in experimental studies, aggression requires instigating conditions [87]. The explanation of the alcohol–aggression link also requires the inclusion of additional factors, such as personality. Researchers have suggested that the alcohol-aggression relationship may be stronger in individuals with preexisting aggressive traits [88]. Support for this hypothesis comes from studies that find individuals with high levels of hostility and trait anger [89], dispositional aggressivity [90], irritability [91], slight cognitive deficits, impulsiveness, exposure to alcoholic parents, economic instability, praising of hyper-masculine identity, membership in a delinquent group in which becoming intoxicated is an eligibility criterion [92,93], and antisocial personality traits [94], are at heightened risk for intoxicated aggression [95]. In contrast, higher levels of dispositional empathy have been shown to be linked with lower levels of aggression [96]. The link between alcohol intoxication and subsequent aggression has been shown to be modulated by trait-aggression. Results from a study by Giancola and colleagues [97] indicated that alcohol increased physical aggression to a much greater extent for persons who had higher, as opposed to lower levels of an aggressive personality.

It is also conceivable that these two aspects are caused by a third as-yet-unknown variable (a psychological one, for example). Fergusson and collaborators [98] tackled this question in a study on the relationship between drinking and violent behavior (aggression and destruction of property) among 15- and 16-year-old adolescents. They found a systematic link between drinking and property destruction, but the association was also due to other risk factors (family structure and socioeconomic status). However, the authors acknowledge that there is probably a cause-and-effect relation between drinking and violent behavior. Rutter [99] recommended that longitudinal studies be conducted to confirm the results obtained by Fergusson and colleagues.

Some definitive answers to this question were offered by MacDonald and colleagues [100] in a study of 30 ERs in six countries. Their study measured the link between the BAC of patients arriving at the ER for a violence-related injury, and persons coming in following an accidental injury. The authors found a link between BAC and violence-related injury. Given that chronologically, the drinking generally comes before the injury, the authors confirmed the idea that alcohol is indeed the cause of behaviors leading to violent acts. Other studies lead by Giancola and colleagues [101] showed similar results.

## 4. Conclusions and Suggestions for Future Research

### 4.1. Developing New Intervention Procedures

Future studies should consider a number of other variables likely to interact with drinking and affect the risk of injury. These include risk perception, at-risk behaviors, and personality variables that might play a role in aggressive behavior and overuse of alcohol. Results from experimental social psychology show that alcohol-related aggressive behavior is also the consequence of internal factors such as expectancies toward alcohol or beliefs in the effect of alcohol on behavior [45,83]. Interventions and prevention programs concerning violent behavior occurring under alcohol intoxication should focus on implicit and explicit (*i.e.*, self-reported) attitudes toward alcohol. Because expectancies interact with alcohol intoxication to increase aggression [102,103,104], expectancy challenging interventions [105] could provide efficient tools to reduce alcohol-related violence. Determining in which cases alcohol plays a causal factor or is merely a consequence of more influent variables is an issue that could only be addressed in an experimental setting. However, studies taking place in the ER should more often take into account psychosocial and personality variables as they could have an impact on alcohol-related aggression [71,105,106]. Prevention programs and interventions could also focus on the patient’s alcohol-related expectancies and personality variables that could have a direct effect on aggressive behavior as well as an indirect effect through alcohol-related expectancies [107]. 

Research from implicit social cognition could also lead to easy-to-implement interventions. For example, recent studies on evaluative conditioning have shown that participants’ attitudes toward alcohol and effective alcohol consumption could be modified by exposing them to alcohol-related images coupled with negatively valenced images by creating an affective transfer between the two stimuli [108,109]. More important about these procedures is that they modify implicit attitude toward a specific object or concept. According to dual-process theory of alcohol addiction, alcohol consumption is mainly guided by automatic memory association and affective reactions toward alcohol [110], thus making procedures tapping into implicit memory more likely to impact those affective reactions and subsequent alcohol consumption. Moreover; changing automatic expectancies toward alcohol could be done with evaluative conditioning procedures. In fact, recent studies have shown that modifying expectancies toward alcohol could lead to significant changes in implicit attitudes toward alcohol and outcome expectancies of drinking alcohol by mere exposure to warning labels [111]. To this day, evaluative conditioning procedure have only been tested in experimental settings and still have to be implemented in ER. More research is needed to assess the impact of changing expectancies by mere exposure on effective alcohol consumption. However, evaluative conditioning should be easy to implement in ER since it is a relatively short procedure (less than 15 min) and do not require trained personnel. 

Recent research has also shown that attribution processes can moderate the link between attitudes and subsequent behavior [112]. Improving the patient’s awareness of the origin of his behavior may improve his ability to control it. More theoretical research on how these phenomena works and how they could apply to ER prevention treatment are still needed. 

### 4.2. Addressing the Current Methodological Limitations in ER Studies

Taking into account personality variables would lead to acknowledge more precisely the characteristics of the samples used in ER studies, thus addressing the sampling issues. Even if it cannot settle the sociodemographic sampling issues that are inherent in ER studies, it would lead to a better understanding of the specific populations which are assessed in the ER for alcohol-related injuries. Moreover, determining whether alcohol is the cause of violence by simply asking patients in the ER might be a complex task. Indeed, research in social psychology has shown that when explaining the behavior of others, people tend to be biased to conclude that the observed behavior was intentional rather than accidental. This intentionality bias (*i.e.*, the tendency for people to view the behavior of others as intentional more often than it really is) has been shown to be bolstered during alcohol intoxication. In a recent study, Bègue and colleagues showed that drunk participants interpreted more acts as intentional than did sober participants [113]. This could lead to difficulties in obtaining reliable answers when interrogating ER patients on the causes of alcohol-related injuries which occurred during an aggression or violent context. Intentionality bias should be integrated into intervention procedure as well. For example, Alcohol Myopia Theory could be used to explain how individuals could reallocate their attentional resources while being intoxicated which leads to impaired appraisal functions concerning other people behavior or intentions and thus be more prone to initiate fights, *etc*. Patients who have been admitted in the ER for an injury resulting from a fight could beneficiate from specific interventions where they will be briefed on the way alcohol biases their perception. 

### 4.3. Improving Existing Procedures

Eventually, an in-depth investigation of psychopathological factors is needed, especially insofar as the type of population that goes to the ER for an alcohol problem is likely to exhibit more psychological disorders than the general population and to have psychological profiles of its own [114]. For example, data shows a high comorbidity between personality disorders such as antisocial personality disorder, affective disorders, traits such as impulsivity and suicidality [115]. A more detailed assessment of personality, psychiatric disorders and alcohol abuse among ER patients could improve detection rates of patients with high probability to commit suicide. From a prevention standpoint, more attention should be paid to the psychological impact of drinking-related injuries and to the impact of emergency hospitalization, such as increasing the risk of suicide after the event which led the patient to the ER. ER practitioners can insist on the particular sensitivity of patients to change regarding their alcohol consumption if they have been admitted to the ER for an alcohol-related injury. If the admission to the ER is related to alcohol intoxication, the interview should be directed towards how to reduce violent behaviors that are caused by alcohol consumption. This implies that the patient should be aware that alcohol is in a way related to the behavior that led him or her to the ER, with the practitioner emphasizing that aspect of drinking and leading the patient to develop a reflexive attitude [26]. Taking expectancies into account could lead to interventions more focused on the patient specific issues. For example, since patients could as well be the victim or the perpetrator of a fight, his or her expectancies toward alcohol and violence may also be “protective”, leading to think that he or she may be stronger or less vulnerable while intoxicated. Such “in-depth” interviews could be used to assess more precisely whether the patient suffers from psychopathological disorders. 

**Table 1:** ER studies measuring self-report of alcohol consumption and BAC among probability samples of injured and violence-related injured patients.

Reference	Year	Location	Alcohol consumption measure	Nb of participants	Injured % positive BAC * (n)	Injured % positive self-report (n)	Injured % w/ violence positive BAC (n)	Injured % w/ violence positive self-report (n)
Cunningham *et al*. [8]	2003	USA	Self-report	320		4,3 (CAGE *)/10,7 (TWEAK *)		15,5 (CAGE)/25,6 (TWEAK)
Cherpitel [116]	1984-85	USA (San Francisco)	BAC	205	14		44	
Borges *et al*. [14]	2001-02	Argentina	Self-report	447	-	17,5	-	48,1
	2001-02	Mexico	Self-report	455	-	10,3	-	51
	2001-02	Brasil	Self-report	489	-	9,7	-	36
Cherpitel & Ye [22]	2002-03	Argentina	Self-report	538	-	25,8	-	57,64
	2002-03	Australia	Self-report	568	-	20,5	-	63,63
	2002-03	Belarus	Self-report	402	-	28,4	-	81,39
	2002-03	Brasil	Self-report	335	-	13,9	-	47,05
	2002-03	Canada	Self-report	447	-	12,7	-	87,09
	2002-03	China	Self-report	209	-	43,6	-	56,86
	2002-03	Czech Republic	Self-report	424	-	7,8	-	31,25
	2002-03	India	Self-report	136	-	70,7	-	84,5
	2002-03	Mexico	Self-report	655	-	23,4	-	62,07
	2002-03	Mozambique	Self-report	269	-	22,5	-	44,92
	2002-03	Poland	Self-report	509	-	8,3	-	63,26
	2002-03	South Africa	Self-report	280	-	72,6	-	86,73
	2002-03	Sweden	Self-report	84	-	13,9	-	60
	2002-03	USA	Self-report	1443	-	19,1	-	54,54
	2002-03	Spain	Self-report	837	-	20,8	-	43,58
MacDonald *et al*. [85]	2005	USA	BAC & Self-report	3272	29,7	50,5	-	-
	2005	Mexico	BAC & Self-report	2587	36,8	49,8	-	-
	2005	Canada	BAC & Self-report	572	61,1	84,9	-	-
	2005	Australia	BAC & Self-report	872	42,7	60,9	-	-
	2005	Spain	BAC & Self-report	1652	25	36	-	-
	2005	Argentina	BAC & Self-report	351	24	47,3	-	-
Weschler *et al*. [12]	1967	USA (Boston)	BAC	5622	23	19	56	-
Peppiatt *et al*. [117]	1976-1977	UK (Exeter)	BAC		10	-	45	-
Walsch & Mcleod [118]	1980	Scotland (Broxburn)	BAC	754	14	-	70	-
Papoz *et al*. [119]	1982-1983	France (Paris & other towns)	BAC	4789	32	-	64	-
Yates *et al*. [120]		UK (Salford)	BAC	1005	14	-	60	-
Cherpitel & Parès [121]	1985	USA (Contra Costa)	BAC	866	9	-	26	-
	1987-1988	Spain (Barcelona)	BAC	1601	10	-	25	-
Rosovsky *et al*. [122]	1986	Mexico (Mexico City)	BAC	1708	12	-	37	-
Cherpitel [14]	1986-1987	USA (Contra Costa)	BAC	1326	14	-	25	-
Cherpitel [19]	1989	USA (Contra Costa)	BAC	367	7	-	17	-
Borgès, Cherpitel & Rosovsky [30]	1986	Mexico (Mexico City)	BAC & Self-report	2507	3,6	7,9 (0,001-100g)	43,2	26,1 (0,001-100g)
Borgès *et al*. [123]	1996	Mexico (Pachuca)	Self-report	127 (ER) 920 (Household)		2,3 (Household)		38,5
Wright & Kariya [25]		Scotland (Paisley)	Self-report	-		15		60
Cherpitel *et al*. [16]		USA (Jackson)	BAC & Self-report	-	7	14	35	55
		USA (Contra Costa)	BAC & Self-report	-	8	11	22	45
MacDonald *et al*. [124]		Canada (Edmonton, Quebec City)	BAC & Self-report	-	9	17	61	84
Peden *et al*. [125]		South Africa (Cape Town)	BAC & Self-report	-	52	36	70	60
Vinson *et al*. [43]		USA (Columbia)	BAC & Self-report	-	9	12	43	48

* BAC: Blood Alcohol Concentration; CAGE: questionnaire for screening alcoholism problems; TWEAK: questionnaire for detecting harmful drinking in the general population.

Certainly, given the high rates of positive BACs and self-reports of alcohol consumption prior to injury among ER patients, routine testing for BAC in the ER should be mandated or highly encouraged in those countries with sufficient resources to implement such a policy, which would be especially important not only clinically, in ruling out intoxication as a co-morbid condition, but also for identifying those who may benefit from a brief intervention or who should be referred.
